# Strong nuclear expression of HOXB13 is a reliable surrogate marker for DNA methylome profiling to distinguish myxopapillary ependymoma from spinal ependymoma

**DOI:** 10.1007/s00401-025-02866-7

**Published:** 2025-03-26

**Authors:** Suvendu Purkait, Sophia Praeger, Jörg Felsberg, David Pauck, Kerstin Kaulich, Marietta Wolter, David Koppstein, Guido Reifenberger

**Affiliations:** 1 Department of Pathology and Laboratory Medicine, All India Institute of Medical Sciences, Bhubaneswar, Odisha India; 2 Institute of Neuropathology, Heinrich Heine University Medical Faculty and University Hospital Düsseldorf, Moorenstraße 5, 40225 Düsseldorf, Germany; 3 Cancer Bioinformatics and Multiomics (ED08), German Cancer Research Center Heidelberg and German Cancer Consortium (DKTK), Partner Site Essen/Düsseldorf, Düsseldorf, Germany; 4 Department of Pediatric Oncology, Hematology and Clinical Immunology, Heinrich Heine University Medical Faculty and University Hospital Düsseldorf, Düsseldorf, Germany; 5 German Cancer Consortium (DKTK), Partner Site Essen/Düsseldorf, Düsseldorf, Germany

**Keywords:** Myxopapillary ependymoma, Differential diagnostics, DNA methylation, HOXB13, Immunohistochemistry, *NF2* mutation

## Abstract

Spinal ependymoma and myxopapillary ependymoma are the two most common spinal ependymal tumor types that feature distinct histological characteristics, genetic alterations and DNA methylation profiles. Their histological distinction may be difficult in individual cases and molecular diagnostic assessment, in particular DNA methylome profiling, may then be required to assign the correct diagnosis. Expression of the homeobox gene *HOXB13* at the mRNA and protein levels has been reported as a frequent finding in myxopapillary ependymoma that may serve as a diagnostic marker for these tumors. Here, we evaluated the diagnostic role of HOXB13 immunostaining in 143 spinal neoplasms, comprising 54 histologically classified myxopapillary ependymomas, 46 histologically classified spinal ependymomas, and various other tumor types. Immunohistochemical results for HOXB13 protein were compared to molecular findings obtained by bead array-based DNA methylation and DNA copy number profiling, as well as next generation gene panel sequencing-based mutational analysis. Our findings indicate strong nuclear HOXB13 expression as a reliable diagnostic marker for molecularly confirmed myxopapillary ependymoma. Moreover, we provide evidence that differential HOXB13 protein expression is related to differential *HOXB13*-associated CpG site methylation in myxopapillary vs. spinal ependymomas, which can be assessed by targeted DNA methylation analysis. Taken together, immunohistochemistry for HOXB13 protein expression and targeted DNA methylation analysis of *HOXB13* represent useful surrogate approaches that may substitute for DNA methylome profiling in routine diagnostics and facilitate precise classification of spinal ependymal tumors. In particular, strong nuclear HOXB13 immunoreactivity may serve as a novel diagnostic criterion for the classification of myxopapillary ependymoma.

**Supplementary Information:**

The online version contains supplementary material available at 10.1007/s00401-025-02866-7.

## Introduction

Ependymal tumors are a heterogeneous group of neuroepithelial neoplasms that may originate along the entire neuroaxis, including the cerebral hemispheres, posterior fossa, and the spinal cord [16, 41]. In pediatric patients, most ependymal tumors are intracranial, while the spinal cord is more commonly affected in adults. Overall, spinal ependymal tumors constitute approximately 18% of all spinal cord tumors across all age groups [38]. Traditionally, the classification and grading of ependymal tumors were based on histology; however, histological diagnostics was found to have limited prognostic implications and was considered insufficient to provide adequate guidance to clinical decision-making [12]. DNA methylation-based classification of ependymal tumors revealed distinct tumor types according to tumor location and molecular characteristics that showed improved associations with tumor biology and clinical outcome [35]. In total, nine distinct types of ependymal tumors were originally identified, including three distinct ependymoma types in each anatomical region (supratentorial, infratentorial and spinal). The three types of spinal ependymal tumors were myxopapillary ependymoma (MPE), spinal ependymoma (SP-EP), and spinal subependymoma (SP-SE). A fourth type of spinal ependymoma was added more recently, namely, spinal ependymoma with *MYCN* amplification, which are highly aggressive tumors characterized by amplification and overexpression of the *MYCN* proto-oncogene [17, 25].

The diagnosis of MPE is primarily based on histological criteria as defined in the 5th edition of the World Health Organization (WHO) classification of central nervous system (CNS) tumors [25]. The classic histology of MPE features radially arranged cuboidal tumor cells around a hyalinized vascular core with variable amounts of perivascular myxoid and microcystic changes. However, histological features in MPE are often heterogeneous and may overlap with classic features in SP-EP, including perivascular pseudorosette formation, hence leading to diagnostic challenges, especially in cases with more solid areas and limited papillary or myxoid differentiation.

Genome-wide DNA methylation profiling has greatly improved diagnostic accuracy for CNS tumors by providing a histology-independent means of brain tumor classification based on tumor type-specific DNA methylation patterns [7]. DNA methylation studies on ependymal tumors have documented that almost all tumors that were histologically diagnosed as MPE shared a common DNA methylation profile that was distinct from all other types of ependymomas and other brain tumors, supporting the specificity of the typical MPE histology when present [32, 35, 48]. However, a significant proportion of spinal tumors histologically diagnosed as SP-EP showed DNA methylation profiles of MPE, indicating that histology-based classification may not be sufficient for diagnostic distinction between MPE and SP-EP [32, 35, 48]. While DNA methylation-based classification is increasingly used, it is not yet globally available, in particular not in resource-restricted countries.

Previous studies indicated upregulated expression of *HOXB13* mRNA and protein in MPE, and proposed nuclear HOXB13 expression as a promising immunohistochemical marker for these tumors [2, 5, 18]. HOXB13 has been implicated in embryonic development, including development of the prostate gland [11]. In addition, strong nuclear HOXB13 expression has been detected in certain types of tumors including prostate carcinoma [22, 36] and cauda equina neuroendocrine tumor (previously paraganglioma) [6]. On the other hand, decreased expression has been detected in gastric and colon carcinomas [43, 50]. Regulation of *HOXB13* transcription and expression has been reported to depend on the activity of the BET domain protein BRD4 and/or differential methylation of *HOXB13*-associated 5’CpG dinucleotides [31, 43, 50]. However, the molecular mechanisms underlying differential expression of HOXB13 in MPE and SP-EP are as yet unknown.

We assessed the diagnostic utility of HOXB13 immunohistochemistry in an institutional cohort of various types of spinal ependymal tumors (*n* = 111) and various other spinal tumor types (*n* = 32). The results of HOXB13 immunostaining were compared to data obtained by bead array-based DNA methylation and DNA copy number profiling as well as next generation gene panel sequencing in selected cases. Moreover, putative epigenetic mechanisms driving *HOXB13* transcription and expression were investigated. Collectively, strong nuclear expression of HOXB13 was validated as a reliable immunohistochemical marker for molecularly confirmed MPE. In contrast, molecularly confirmed SP-EP and all other types of spinal ependymal tumors lacked strong nuclear HOXB13 expression. Among the non-ependymal spinal tumor types, only cauda equina neuroendocrine tumor (previously paraganglioma) demonstrated strong nuclear HOXB13 expression. We further present evidence that the upregulation of HOXB13 in MPE (and in cauda equina neuroendocrine tumors) is likely due to differential *HOXB13*-associated CpG site methylation when compared to SP-EP.

## Materials and methods

### Patients’ samples

Formalin-fixed and paraffin-embedded (FFPE) tumor tissue samples were retrieved from the archive of the Institute of Neuropathology, Heinrich Heine University and University Hospital Düsseldorf, Germany. In total, tumor tissue samples from 143 patients with different types of spinal tumors, consisting of 111 ependymal tumors, including myxopapillary ependymoma (MPE), spinal ependymoma (SP-EP), spinal subependymoma (SP-SE), spinal ependymoma, *MYCN*-amplified (SP-EP-MYCN), and spinal manifestations of posterior fossa ependymoma, group A or B (PFA-EP, PFB-EP), 32 non-ependymal spinal tumor types, as well as 3 non-neoplastic spinal cord tissue samples obtained at autopsy were investigated in the study (Table 1, Suppl. Table 1). Among the ependymal tumors, most were from adult patients (*n* = 103), while 8 tumors (5 MPE, 1 SP-EP and 2 PFA-EP spinal metastases) were from patients younger than 18 years at the time of tumor resection.

**Table 1:** Baseline characteristics of the spinal tumors investigated for HOXB13 expression

Diagnosis	CNS WHO grade	Number of patients	Age at diagnosis [years, mean (range)]	Gender (M:F)
Myxopapillary ependymoma*	2	54	46 (8–87)	1.4:1
Myxopapillary ependymoma**	2	65	48 (8 87)	1.5:1
Spinal ependymoma*	2	46	49 (7–72)	0.9:1
Spinal ependymoma**	2	35	47 (7–72)	1.2:1
Spinal subependymoma	1	5	42 (28–71)	1:4
Spinal metastasis of posterior fossa ependymoma, group A or B	3	4	30 (15–46)	1:1
Spinal ependymoma, *MYCN*-amplified	n.d	2	71 (56–86)	1:1
Spinal diffuse midline glioma, H3 K27-altered	4	3	35 (26–50)	2:1
Spinal pediatric type diffuse high-grade glioma, H3-wildtype and IDH-wildtype	4	1	3	-:1
Spinal glioblastoma, IDH-wildtype	4	1	66	-:1
Spinal pilocytic astrocytoma	1	5	27 (11–63)	1:1.5
Spinal meningioma	1 or 2	6	71 (48 -85)	1:2
Spinal schwannoma and neurofibroma	1	6	57 (31–74)	2:1
Spinal melanotic peripheral nerve sheath tumor	n.d	1	59	1:-
Spinal meningeal melanocytoma/melanocytic tumor of intermediate differentiation	n.d	4	55 (41–77)	4:-
Cauda equina neuroendocrine tumor (formerly paraganglioma)	1	5	52 (34–63)	4:1

*Original histology-based diagnoses without consideration of HOXB13 immunohistochemistry;

**Revised diagnoses considering HOXB13 immunohistochemistry and DNA methylome profiling (if available)

*M* male; *F* female -; no patients of the particular gender; n.d., CNS WHO grade not defined

All patients had been diagnosed and treated at the University Hospital Düsseldorf between 2009 and 2024. The respective tissue samples and associated patient data were used in an anonymized manner as approved by the Institutional Review Board of the Medical Faculty, Heinrich Heine University Düsseldorf for the use of archival tissue samples for research purposes (study number 3562). In addition, a project-specific ethics vote was obtained from the Institutional Review Board for this study (study number 2024–2841).

### Histology and immunohistochemistry

All tumors were histologically analyzed on hematoxylin–eosin (H&E)-stained sections and classified according to the 5th edition of the World Health Organization (WHO) classification of central nervous system (CNS) tumors [25]. Mucin was demonstrated by alcian blue staining. Immunohistochemistry was performed on FFPE tissue sections using a Dako Autostainer Link 48 immunostainer (Agilent Technologies, Santa Clara, CA). The following primary antibodies were used for immunohistochemistry: anti-HOXB13 mouse monoclonal antibody F9 (Santa Cruz Biotechnology, Dallas, Tx; sc-28333; dilution 1:200), anti-BRD4 rabbit monoclonal antibody (Abcam, Cambridge, UK; ab128874; dilution 1:200), anti-glial fibrillary acidic protein (GFAP) rabbit monoclonal antibody (ZETA Corporation, Monrovia, CA; ZR356; dilution 1:100), anti-epithelial membrane antigen (EMA) mouse monoclonal antibody E29 (Agilent Technologies; dilution 1:500), and anti-MYCN rabbit monoclonal antibody D4B2Y (Cell Signaling Technology Inc., Danvers, MA, dilution 1:100). Antigen binding of the primary antibodies was detected with the EnVision FLEX system (Agilent Technologies) using 3,3-diaminobenzidine as horseradish peroxidase substrate and chromogen. Sections were counterstained with hemalum. Nuclear expression for HOXB13 was categorized into “strong”, “weak” or “negative” immunostaining. Nuclear HOXB13 expression was considered as “strong” when a dark brown nuclear labelling was detected in tumor cells. HOXB13 positivity was considered as “weak” when only a faint nuclear immunostaining was observed in tumor cells. Tumors were considered as “negative” when no nuclear HOXB13 positivity was present in tumors cells.

### DNA extraction from FFPE tumor tissue samples

Tumor DNA was extracted from FFPE tissue samples using the QIAamp DNA FFPE Advanced kit (Qiagen, Hilden, Germany) according to the manufacturer’s protocol. Extracted DNA was quantified spectrophotometrically using the Quantus^™^ fluorometer (Promega, Madison, WI). Only tissue specimens with a histologically estimated tumor cell content of 80% or more were used for DNA extraction. In a subset of cases, microdissection was used to obtain tumor cell areas with ≥ 80% tumor cell content for DNA extraction.

### ***Infinium***^***™***^*** methylation EPIC v2.0 bead chip-based DNA methylation profiling***

Twenty-nine selected cases of spinal ependymal tumors (Suppl. Table 2) were subjected to DNA methylation profiling using hybridization of Infinium^™^ methylation EPIC v2.0 bead chip (Illumina, San Diego, CA). DNA methylation data were analyzed with the Heidelberg brain tumor classifier algorithm version v.12.8 (www.molecularneuropathology.org), and the tumors were accordingly assigned to the methylation classes “myxopapillary ependymoma (MPE)”, “spinal ependymoma (SP-EP)”, “spinal subependymoma (SP-SE)”, and others based on calibrated classifier scores. In addition to DNA methylation-based class assignment, genome-wide DNA copy number information was derived from the Infinium^™^ methylation EPIC v2.0 data set. The principles of the DNA methylation-based classification of CNS tumors, the assignment of tumors to distinct methylation classes, the role of the calibrated classified score, and the generation of copy number profiles from the methylation data set have been described in detail before [7].

### Bioinformatics evaluation for differential CpG site methylation in HOXB13

The EPIC v2.0 methylation array data were analysed following the cross-package Bioconductor workflow for analysing methylation array data [15, 26]. Only patients whose tumors demonstrated a calibrated score of > 0.9 were considered, resulting in nine SP-EP and eleven MPE patients. The methylation data were processed and annotated with an EPIC v2.0 probe manifest corresponding to the GRCh38 genome [19, 20] using the minfi package [1, 14]. The data were subjected to stratified quantile normalization [45] and probes on sex chromosomes, probes with SNPs at CpG sites, and probes that failed in at least one sample were excluded from the analysis. For each CpG site and patient, the *M* values and beta values were calculated as $\text{M = log(}\frac{\text{Meth }}{\text{Unmeth}}\text{)}$ and $\beta = \frac{\text{Meth}}{\text{(Meth+Unmeth+100)}},$ where ${\text{Meth}}$ refers to the methylated and ${\text{Unmeth}}$ to the unmethylated intensity [26]. A linear model was fitted on the *M* value matrix using the limma package [40] and contrasts were set based on disease entity. Each CpG dinucleotide was tested for differential methylation between the two tumor groups using the moderated t-statistic [42]. Multiple testing was controlled with the Benjamini–Hochberg procedure [3]. A CpG site with adjusted *p* value < 0.05 was considered as significantly differentially methylated between the two groups. The beta values of CpG sites that map to *HOXB13* were converted to a GRanges object [24] and plotted using the package Gviz [21, 33] with Ensembl GRCh38 annotation [9, 10, 28] in the style of DMRcate [37]. The genomic positions of the CpG islands associated with *HOXB13* were taken from the UCSC Genome Browser [30; https://genome.ucsc.edu/]. The data analysis was orchestrated with a custom R script, version 4.4.1 [29, 39, 47] and is available from the corresponding author upon request.

### Targeted analysis of *HOXB13* methylation using sodium bisulfite pyrosequencing

The methylation status of the CpG site cg01799458_BC21 located in the first intron of *HOXB13* (chr17:48,727,442) was determined by DNA pyrosequencing of sodium bisulfite-modified DNA using the PyroMark^®^ Q24 platform (Qiagen, Hilden, Germany). This CpG site was selected for targeted methylation analysis because of a significantly lower methylation level in myxopapillary compared to spinal ependymoma determined by analysis of our EPICv2 array-based data set. We investigated 70 tumors (including 65 adult and 5 pediatric tumors) that were studied for HOXB13 expression by immunohistochemistry (39 HOXB13-positive MPE, 4 HOXB13-positive cauda equina neuroendocrine tumors, 25 HOXB13-negative SP-EP, and 2 HOXB13-negative SP-EP-MYCN) by pyrosequencing for methylation of the CpG site cg01799458_BC21. In total, 200 ng of DNA from each tumor was treated with sodium bisulfite using the MethylEdge® Bisulfite Conversion System (Promega, Walldorf, Germany). The primer sequences used to amplify bisulfite-converted DNA surrounding the cg01799458_BC21 site were cg01799458_forward 5′-GGTTATTTTTTTAGATTTTATAGGTAAATTTTG and cg01799458_reverse 5′-[Btn] CCAAAAAAAAATTTAAATTTCCTACAACC. The primers amplify a 151 bp fragment. Pyrosequencing was done with 0.3 µM of cg01799458_Seq 5′-GTTTTTGTTTTTATTTTTAATATATG. PCR was performed using standard conditions with 2 µl of sodium bisulfite-converted DNA as template in a total volume of 25 μl and HotStar-*Taq*-DNA polymerase (Qiagen). An initial 15 min activation of the *Taq* polymerase at 95 °C was followed by 45 cycles at 95 °C for 30 s, 54 °C for 30 s, and 72 °C for 30 s, with a terminal elongation step at 72 °C for 5 min. 20 µl of each PCR product was used for pyrosequencing according to the manufacturer´s protocol (Qiagen, PyroMark^®^ Q24 User Manual, Version 5, January 2016).

### Glioma gene-panel next generation sequencing

The 29 tumors subjected to microrray-based DNA methylation profiling were additionally investigated by amplicon-based gene panel next-generation sequencing using a previously reported customized glioma-associated gene panel [51]. The gene panel consisted of 660 primer pairs and covered the entire coding sequence (cds) or mutational hotspots of 20 glioma-associated genes, namely, *ATRX* (cds), *BRAF* (hot spot region), *CDKN2A* (cds), *CDKN2B* (cds), *CDKN2C* (cds), *CIC* (cds), *EGFR* (cds), *FUBP1* (cds), *H3-3A* (hot spot region), *IDH1* (hot spot region), *IDH2* (hot spot region), *NF1* (cds), *NF2* (cds), *NRAS* (cds), *PIK3CA* (cds), *PIK3R1* (cds), *PTEN* (cds), *RB1* (cds), *TERT* promoter (hot spot region) and *TP53* (cds). We used this glioma-associated gene panel as it covers the entire coding sequence of the *NF2* gene that is commonly altered by monoallelic deletion combined with mutations in a subgroup of spinal ependymomas [34]. NGS libraries were constructed with the Ion AmpliSeqTM Library 2.0 Kit (Life Technologies) and ten nanograms of genomic tumor DNA per primer pool. Sequencing was performed on the Ion S5 XL system (Thermo Fisher Scientific, Waltham, MA). The DNA sequences obtained for the covered genes were aligned to the human reference genome assembly GRCh37 (hg19) (https://www.ncbi.nlm.nih.gov/assembly/2758) to detect sequence variations.

### Detection of *MYCN* amplification by droplet digital PCR

Amplification of *MYCN* in the two cases of SP-EP-MYCN was demonstrated by droplet digital PCR using the PrimePCR™ *MYCN* ddPCR copy number assay dHsaCP2500435 (Bio-Rad, Feldkirchen, Germany) with two reference loci as controls [49].

### Statistical analysis

The difference in the frequency of the chromosomal copy number alterations between MPE and SP-EP were analyzed using Fisher’s exact test. The difference in methylated allele frequency at cg01799458 between MPE and SP-EP was analyzed using a two-tailed unpaired *T* test. A *p* value < 0.05 was considered as statistically significant.

## Results

### HOXB13 protein expression in spinal ependymal tumors

**Fig. 1:**
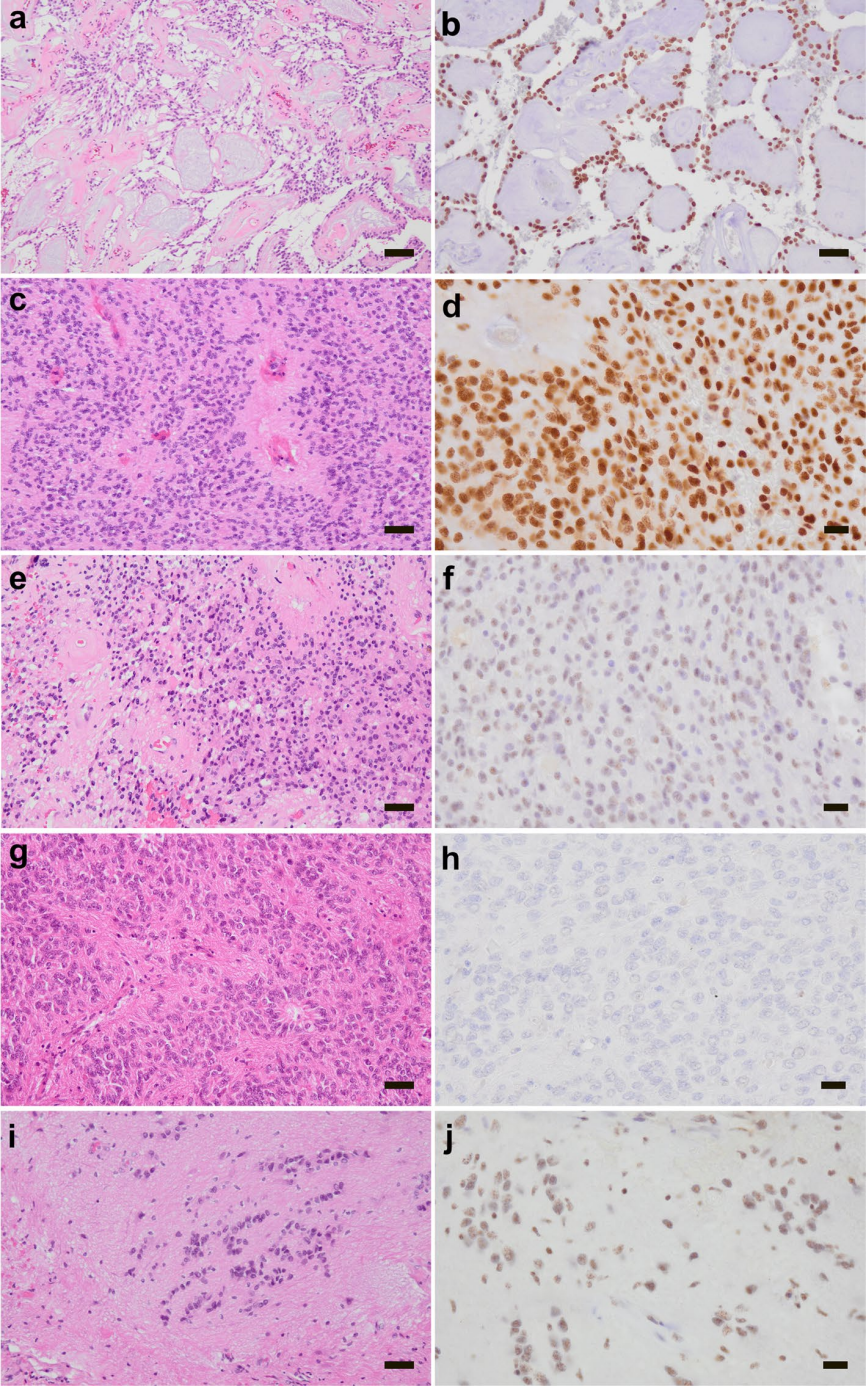
Histological features and immunohistochemical staining patterns for HOXB13 in selected cases of spinal ependymal tumors. **a**, **b** MPE (case MPE54) with papillary configuration and abundant myxoid areas (**a**) as well as strong nuclear expression of HOXB13 (**b**). **c**, **d** Histologically diagnosed spinal ependymoma (case E176) with typical perivascular pseudorosettes and lack of myxopapillary features (**a**), but strong nuclear expression of HOXB13 (**d**). **e**, **f** Another spinal ependymoma (case E175) with classic ependymoma histology (**e**) and weak nuclear positivity for HOXB13 (**f**). **g**, **h** Spinal ependymoma (case E77) with classical histology including ependymal rosette and perivascular pseudorosette formation (**g**) as well as complete lack of HOXB13 immunostaining (**h**). **i**, **j** Spinal subependymoma (case SE110) with clustering of tumor cell nuclei within a fibrillar matrix (**i**) and weak nuclear expression of HOXB13 (**j**). Sections in **a**, **c**, **e**, **g** and **i** are stained with hematoxylin and eosin. Sections in **b**, **d**, **f**, **h** and **j** are immunohistochemically stained with the anti-HOXB13 antibody (brown) and counterstained with hemalum (blue). Scale bars correspond to 20 mm (**d**, **f**, **h**, **j**), 50 mm (**b**, **c**, **e**, **g**, **i**), or 100 mm (**a**)

Immunohistochemistry for HOXB13 protein expression was performed on a cohort of 143 spinal tumors and three non-neoplastic spinal cord tissue samples (Table 1, Suppl. Table 1). Among the 111 investigated ependymal tumors, we observed three distinct patterns of HOXB13 immunostaining, namely, strong nuclear immunoreactivity (dark brown staining in the vast majority or all tumor cells), weak nuclear immunoreactivity (faint staining of variable numbers of tumor cells), and no nuclear immunoreactivity (Fig. 1). All 54 histologically classified cases of MPE (including 5 MPE from pediatric patients) and all 5 cauda equina neuroendocrine tumor (previously paraganglioma) showed strong nuclear expression of HOXB13 (Fig. 1a, b; Suppl. Figure 1b). In addition, 11/46 cases of histologically classified SP-EP (24.0%) also showed strong nuclear HOXB13 immunoreactivity (Fig. 1c, d), similar to the MPE and cauda equina neuroendocrine tumor cases. Another 10 SP-EP showed weak nuclear HOXB13 immunoreactivity (Fig. 1e, f). The remaining 25 cases of SP-EP (60.0%), including the single pediatric SP-EP, stained uniformly negative for HOXB13 (Fig. 1g, h). Three of 5 cases of SP-SE also contained cells with weak nuclear immunopositivity for HOXB13 (Fig. 1i, j), while the other two SP-SE were negative for HOXB13. The two SP-EP-MYCN tumors (Suppl. Figure 2) and all four spinal metastases of posterior fossa ependymomas lacked nuclear HOXB13 immunoreactivity. None of the other investigated spinal tumors, including different types of low-grade and high-grade astrocytic gliomas, meningiomas, Schwann cell tumors, and melanocytic tumors showed nuclear HOXB13 positivity (Suppl. Table 1, Suppl. Figure 1c–h). Similarly, immunostaining of non-neoplastic adult spinal cord tissue samples obtained at autopsy (n = 3) lacked immunoreactivity for HOXB13 (Suppl. Figure 1a). Overall, we did not notice differences in HOXB13 immunostaining according to age of the respective archival tissue blocks that were stored for up to 15 years (2009–2024). To additionally assess stability of nuclear HOXB13 expression after prolonged fixation in formalin, we investigated tissues samples from one selected MPE after routine formalin fixation for 12 h and after prolonged formalin fixation for 6 weeks, which revealed no difference in staining intensity indicating that nuclear HOXB13 staining in MPE remains stable even after prolonged fixation time (Suppl. Figure 3).

### EPIC v2.0 bead array-based DNA methylation profiling

In total, spinal ependymal tumors of 29 patients were subjected to DNA methylation and copy number profiling using Infinium™ methylation EPIC v2.0 bead chip technology and the Heidelberg classifier for central nervous system tumors (www.molecularneuropathology.org). The investigated tumors included 6 SP-EP with strong nuclear HOXB13 expression, 6 SP-EP with weak nuclear HOXB13 expression, 4 SP-EP without nuclear HOXB13 expression, 6 SP-MPE with strong nuclear HOXB13 expression (mostly cases containing areas with classic ependymoma features in addition to typical myxopapillary parts), 3 SP-SE with weak or absent nuclear expression of HOXB13, and 4 spinal metastases of posterior fossa ependymoma.

All cases of MPE, including those with ependymoma-like areas, were assigned to the methylation class of MPE, in 5/6 tumors with a calibrated classifier score of > 0.9 (Suppl. Table 2). In addition, all six cases of SP-EP with strong nuclear HOXB13 expression were assigned to the MPE methylation class with calibrated classifier scores of > 0.9. On the other hand, none of the 10 SP-EP with either weak or absent nuclear HOXB13 expression, the 3 SP-SE with weak or negative nuclear HOXB13 expression, and the 4 HOXB13-negative spinal metastases of posterior fossa ependymoma was assigned to the MPE methylation class. The latter 4 tumors corresponded to spinal metastases of 3 PFB-EP and 1 PFA-EP cases (Suppl. Table 2).

DNA copy number profiles obtained from the EPICv2 data showed losses of 22q in all 10 cases of SP-EP showing either weak or absent nuclear HOXB13 expression, but only 2 of 12 cases of molecularly confirmed MPE with strong HOXB13 positivity (p = 0.0001, Fisher’s exact test). In contrast, gains of whole chromosome 16 were observed in 5/12 of the strongly HOXB13-positive ependymal tumors corresponding to the MPE methylation class subsets, while none of the 10 molecularly confirmed SP-EP had gains on chromosome 16 (*p* < 0.05, Fisher’s exact test) (Suppl. Table 2, Suppl. Figure 4d). None of the 12 tumors with strong nuclear HOXB13 expression showed evidence of high-level copy number gain/amplification of the *HOXB13* locus on 17q21.32, and only a single HOXB13-immunopositive tumor demonstrated a whole chromosome 17 low-level copy number gain (Suppl. Table 2). These findings thus argue against *HOXB13* copy number gain/amplification as a likely mechanism driving HOXB13 expression in these tumors. DNA copy number profiles of selected ependymal tumors and the cumulative copy number profiles of HOXB13-positive MPE vs. HOXB13-negative SP-EP are shown in Suppl. Figure 4a–d.

**Fig. 2:**
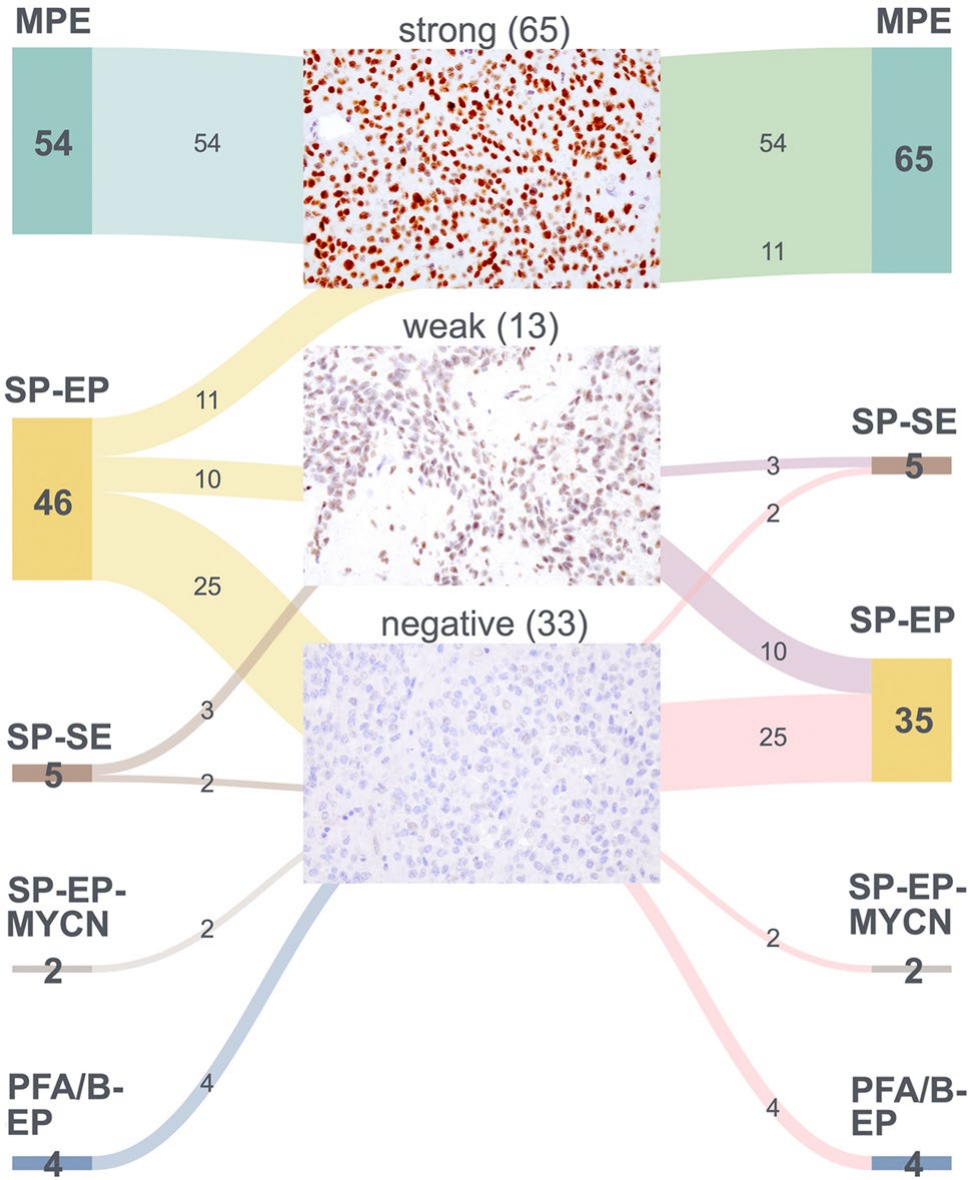
Alluvial plot depicting the effect of HOXB13 immunohistochemistry on the diagnosis and distribution of diagnoses assigned to spinal ependymal tumors classified by histology only or by histology and HOXB13 immunohistochemistry. The switch of diagnosis of the spinal ependymomas (SP-EP) with strong HOXB13 expression to myxopapillary ependymoma (MPE) was confirmed in 6/6 cases investigated by DNA methylation profiling (Suppl. Table 2). Similarly, the retention of the final diagnoses of SP-EP or spinal subependymoma (SP-SE) in cases with only weak HOXB13 expression was molecularly confirmed in all of these tumors additionally investigated by DNA methylation profiling (Suppl. Table 2). SP-EP-MYCN, spinal ependymoma, *MYCN*-amplified; PFA/B-EP, spinal metastases from PFA or PFB ependymomas

Overall, the sensitivity and specificity of strong nuclear HOXB13 immunopositivity for the assignment of tumors to the MPE methylation class in our cohort was 100% each, i.e., all the cases with strong nuclear HOXB13 expression were assigned to the methylation class of MPE, while all of the cases that lacked strong nuclear HOXB13 staining were assigned to the methylation classes of SP-EP, SP-SE or PFA/B-EP. When classification was based on histology, strong nuclear HOXB13 immunopositivity had a sensitivity of 100% (54/54 cases) for detection of histologically classified MPE. However, specificity was lower, as 11/46 (24%) histologically diagnosed SP-EP also stained strongly positive for HOXB13. The changes from the initial histology-based diagnoses to the diagnoses considering HOXB13 nuclear staining (corresponding to the DNA methylation class assignment) are summarised in Fig. 2.

### Gene-panel next generation sequencing

The 29 cases of spinal ependymal tumors investigated by EPICv2-based DNA methylation profiling were additionally subjected to glioma gene panel NGS, in particular to assess their *NF2* gene mutation status. None of the cases with strong nuclear HOXB13 expression and assignment to the MPE methylation class carried *NF2* variants. An *EGFR* missense mutation was detected in a single MPE (Suppl. Table 2). Among the 10 SP-EP with weak or absent nuclear HOXB13 immunoreactivity and assignment to the SP-EP methylation class, *NF2* variants were detected in two tumors (20%) (Suppl. Table 2). A missense mutation in *RB1* was detected in a single case of SP-EP, while one SP-SE carried a *TERT* promoter variant (Suppl. Table 2).

### Morphological re-evaluation of the SP-EP cases with strong nuclear HOXB13 expression

We histologically reassessed the 11 cases of SP-EP with strong HOXB13 expression, including the 6 tumors assigned to the MPE methylation class based on DNA methylation profiling. None of these cases showed histological features indicative of MPE. However, seven tumors showed rare foci of myxoid change. Such focal myxoid areas were, however, also detected when carefully re-assessing the histology of the SP-EP cases with weak or negative nuclear HOXB13 expression (including the cases assigned to the SP-EP methylation class) in 14 of 35 cases (Suppl. Figure 5a–f). On the other hand, a subset (14/54, 26%) of the histologically typical cases of MPE also demonstrated solid areas of classic ependymoma-like histology that showed strong nuclear HOXB13 immunoreactivity (Suppl. Figure 6a–f).

### Immunohistochemistry for BRD4 protein expression

The BET domain containing protein BRD4 has been implicated as a regulator of *HOXB13* transcription that can bind to an enhancer region in *HOXB13* and thereby drive transcriptional upregulation [31]. To screen for possible differential expression of BRD4, we performed immunohistochemistry for BRD4 in selected cases of MPE with strong nuclear HOXB13 expression and SP-EP with lack of nuclear HOXB13 staining. Both tumor types showed identical strong and uniform BRD4 expression (Suppl. Figure 7a, b), thus excluding differential BRD4 expression as a likely mechanism driving differential HOXB13 expression in these two tumor types.

### Detection of differential CpG site methylation in *HOXB13* between SP-EP and MPE based on EPIC v2.0 methylation profiling and targeted sodium bisulfite pyrosequencing.

**Fig. 3:**
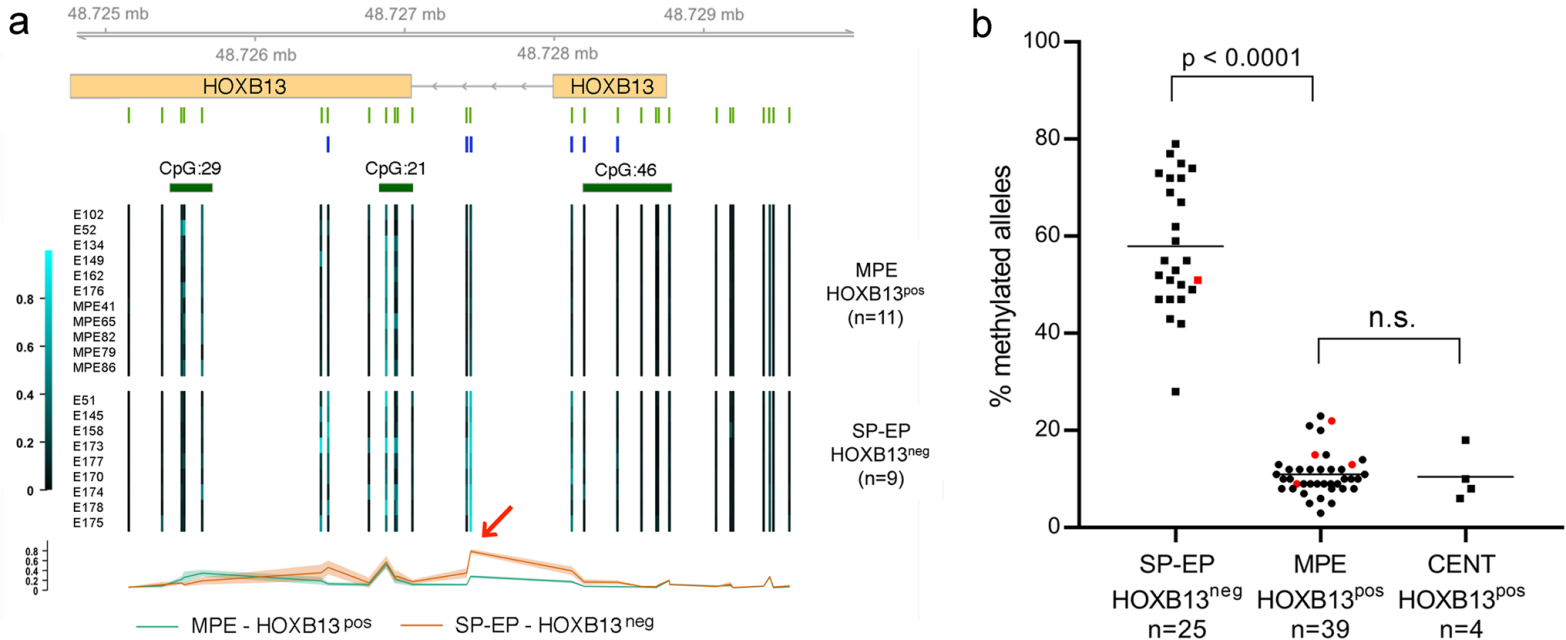
**a** Differential methylation of CpG sites mapping to *HOXB13* as detected by DNA methylation profiling. Beta values of CpG site methylation assessed by EPIC v2.0 analysis are presented for each individual patient as a heatmap and are separated by tumor type (MPE, *n* = 11 vs. SP-EP, *n* = 9). The average beta value for each CpG site according to tumor type is presented at the bottom with a confidence interval of 0.3. Annotated CpG islands from the UCSC Genome Browser are plotted in dark green. Individual CpG sites represented on the EPIC v2.0 bead array are indicated as green bars, and CpG sites showing significant differential methylation between the two ependymoma types are indicated as blue bars. The red arrow points to CpG site cg01799458 that shows the most pronounced differential methylation. **b** Significant difference in the DNA methylation level (indicated by per cent of methylated alleles) of the *HOXB13*-associated CpG site cg01799458 as detected by sodium bisulfite pyrosequencing in strongly HOXB13-positive MPE (*n* = 39) and 4 cauda equina neuroendocrine tumors (formerly paragangliomas) (CENT) as compared to HOXB13-negative or only weakly positive SP-EP (*n* = 25). Red dots indicate pediatric cases

To assess whether the differential HOXB13 protein expression between SP-EP and MPE is related to differential methylation of CpG sites associated with the *HOXB13* gene locus, EPIC v2.0 methylation array results were compared between nine SP-EP and eleven MPE from our spinal ependymoma cohort. We identified 56,503 differentially methylated CpG sites distributed across the genome (moderated *t* test, B–H corrected, p.adj < 0.05), of which six mapped to the *HOXB13* locus (Fig. 3a). The beta values corresponding to the relative frequency of methylation were calculated for each CpG site. Based on these results, the intronic probe cg01799458-BC21 (chr17:48,727,442), which displayed average beta value differences of 0.510 between SP-EP and MPE, was chosen for independent validation using pyrosequencing of sodium bisulfite-modified tumor DNA. We thereby confirmed that this *HOXB13*-associated CpG site is strongly methylated in SP-EP (mean methylated allele frequency: 58.0%, SD: 13.3%) but showed significantly lower methylation levels in MPE (mean methylated allele frequency: 11.0%, SD: 4.5%) (Fig. 3b). Hence, targeted methylation analysis of this CpG site in *HOXB13* by sodium bisulfite pyrosequencing may serve as an alternative molecular approach to bead-array-based DNA methylation profiling for molecular distinction of MPE from SP-EP. Similar to MPE, cauda equina neuroendocrine tumors with strong nuclear HOXB13 positivity showed low levels of cg01799458-BC21 methylation (mean methylated allele frequency: 10.5%, SD: 5.3%) (Fig. 3b).

## Discussion

Myxopapillary ependymoma (MPE) is an ependymal tumor that predominantly occurs in adults and is almost exclusively located in the conus medullaris and filum terminale region of the spinal cord. The cell of origin of these tumors is unknown and the molecular alterations that drive their tumorigenesis are as yet poorly understood. Most MPE follow an indolent clinical course but long-term follow-up data indicate a propensity towards local recurrence and rare metastatic potential [23, 46]. Thus, MPE is considered as a CNS WHO grade 2 tumor in the 5th edition of the WHO classification of CNS tumors [25]. WHO classification of MPE is primarily based on histological features, with essential diagnostic criteria being defined as a “*glioma with papillary structures and perivascular myxoid change or at least focal myxoid microcysts AND immunoreactivity for glial fibrillary acidic protein (GFAP)*” [25]. In addition, MPE have been reported to carry a distinct DNA methylation profile [5, 7, 35, 48], hence the WHO classification also considers a DNA methylation profile aligned with MPE as an essential criterion for diagnosis of histologically unresolved lesions [25]. However, studies on spinal ependymal tumors have shown that a proportion of histologically defined spinal ependymomas (SP-EP) that lack obvious myxopapillary features upon histology are assigned to the MPE methylation class upon DNA methylation profiling [7, 35, 48]. Immunohistochemical studies reported on strong nuclear HOXB13 expression in MPE, while other types of ependymoma and spinal astrocytomas showed only weak or absent HOXB13 positivity [2, 18]. Increased levels of *HOXB13* mRNA have also been documented in MPE [7, 35]. Bockmayr et al. [5] reported on nuclear immunoreactivity for HOXB13 in 97.4% (75/77) of spinal ependymal tumors assigned to the MPE methylation class upon array-based DNA methylation analysis, irrespective of the assignment to either of the two MPE methylation subclasses identified by these authors. On the other hand, no nuclear HOXB13 expression was detected in 15 SP-EP, four spinal subependymomas (SP-SE), five spinal ependymomas with *MYCN* amplification (SP-EP-MYCN), four spinal diffuse midline glioma with H3 K27 alteration, four spinal pilocytic astrocytomas, and one rosette-forming glioneuronal tumor. In the present study, we confirm these findings in an independent institutional cohort of 143 patients with spinal cord tumors, including 111 ependymal tumors, and provide further evidence that strong nuclear HOXB13 immunostaining can distinguish MPE confirmed by DNA methylation profiling from the other types of spinal ependymal tumors with high sensitivity and specificity. Moreover, we also document that weak nuclear HOXB13 expression, as detected in subsets of SP-EP and SP-SE, should not be considered as diagnostic of MPE. A previous study also reported on weak HOXB13 expression in individual cases histologically diagnosed as MPE or supratentorial ependymoma; however, the results were not related to DNA methylation-based classification [2]. Taken together, strong and widespread nuclear HOXB13 immunoreactivity can be regarded as a reliable diagnostic marker. In case of doubt due to weak or only focal HOXB13 immunostaining, either targeted *HOXB13* methylation analysis by DNA pyrosequencing or global DNA methylation profiling may be used to establish the correct diagnosis.

In addition to spinal ependymal tumors, we also evaluated immunohistochemical expression of HOXB13 in selected cases representing other types of primary spinal cord tumors. In line with other reports [5, 6], strong immunoreactivity was identified in cauda equina neuroendocrine tumors but not in different types of spinal astrocytic gliomas, schwannomas, meningiomas or melanocytic tumors. The differential diagnosis between the two HOXB13-positive spinal tumor types, namely, MPE and cauda equina neuroendocrine tumor, can readily be achieved in the routine setting based on typical histological features and distinctive immunohistochemical staining patterns for glial and neuroendocrine markers.

Expression of HOXB13 plays an important role in developmental processes, such as the proper development of the prostate [11]. In addition, aberrant expression of HOXB13 has been reported in various types of cancers outside the CNS, including prostate, colorectal, breast and ovarian carcinomas [4]. Furthermore, a *HOXB13* germline variant has been associated with increased risk for developing prostate cancer [13]. The functional roles of HOXB13 in driving tumorigenesis are likely tumor-specific, with both oncogenic or tumor-suppressive functions being reported [8, 50].

With respect to regulation of HOXB13 expression, the Bromodomain and Extra-Terminal (BET) domain containing protein BRD4 has been shown to bind to the *HOXB13* enhancer and thereby cause its transcriptional upregulation [31]. We performed BRD4 immunohistochemistry in selected cases of HOXB13-positive MPE and HOXB13-negative SP-EP but found uniformly strong and widespread nuclear BRD4 expression in both tumor types (Suppl. Figure 5a, b), indicating that BRD4 expression may not be responsible for differential HOXB13 expression in spinal ependymal tumors.

A recent study revealed frequent copy number gain or amplification of *HOXB13*, including co-amplification with *ERBB2*, as a mechanism driving increased HOXB13 expression in a subset of breast cancer [27]. However, DNA copy number profiling of MPE with strong nuclear HOXB13 expression did not reveal any evidence for HOXB13 copy number gains except for a single case with a low-level copy number gain of the entire chromosome 17.

Various other studies have linked transcriptional regulation of *HOXB13* to differential CpG site methylation in the *HOXB13* promoter region or gene body in different types of cancers, including prostate cancer [36], gastric carcinomas [43], and breast cancer [44]. Based on the DNA methylation profiles in our cases of MPE and SP-EP, we were able to demonstrate that different *HOXB13*-associated CpG sites were commonly strongly methylated in SP-EP but not in MPE, thus pointing to a role of epigenetic regulation of *HOXB13* transcription and protein expression in these tumors. We further validated differential CpG site methylation in *HOXB13* using targeted sodium bisulfite pyrosequencing of the CpG site cg01799458_BC21, which showed clear evidence for differential methylation between SP-EP and MPE upon EPICv2-based DNA methylation profiling. Further studies would be required to clarify the precise mechanisms how reduced *HOXB13* CpG site methylation may lead to transcriptional upregulation of the gene. Nevertheless, our results support differential DNA methylation of *HOXB13* between SP-EP and MPE and suggest targeted *HOXB13* methylation analysis as a further molecular test option to distinguish between the two tumor types in the diagnostic setting, e.g., when immunohistochemistry for HOXB13 is inconclusive.

In summary, the data reported in this study together with previous findings [2, 5, 18] support that strong nuclear HOXB13 expression distinguishes MPE from SP-EP and SP-SE, as well as other spinal tumors except for cauda equina neuroendocrine tumor (previously paraganglioma). Our data, furthermore, show that differential DNA methylation of *HOXB13* is a likely mechanism underlying the differential HOXB13 protein expression in MPE and cauda equina neuroendocrine tumors vs. SP-EP. Concerning diagnostic assessment of spinal ependymal tumors, we propose strong nuclear HOXB13 immunostaining as a valuable and easy to assess surrogate marker for DNA methylation profiling that may serve as an additional diagnostic criterion for MPE.

## Supplementary Information

Below is the link to the electronic supplementary material.

Supplementary file1 (PDF 10824 KB)

## Data Availability

The datasets from this study are available from the corresponding author on reasonable request.

## Abbreviations

CENT: Cauda equina neuroendocrine tumors (previously paraganglioma)
CNS: Central nervous system
MPE: Myxopapillary ependymoma
PFA-EP: Posterior fossa ependymoma, group A
PFB-EP: Posterior fossa ependymoma, group B
SP-EP: Spinal ependymoma
SP-SE: Spinal subependymoma
SP-EP-MYCN: Spinal ependymoma,* MYCN*-amplified
WHO: World Health Organization
