# Supplementary material for: Strong nuclear expression of HOXB13 is a reliable surrogate marker for DNA methylome profiling to distinguish myxopapillary ependymoma from spinal ependymoma
Source: Acta Neuropathol. 2025 Mar 26;149(1):29. doi: 10.1007/s00401-025-02866-7 (PMC11947044; doi:10.1007/s00401-025-02866-7)
Supplement: Supplementary file 1 — Supplementary file1 (PDF 10824 KB) [file 401_2025_2866_MOESM1_ESM.pdf]

Strong nuclear expression of HOXB13 is a reliable surrogate marker for DNA methylome profiling to distinguish myxopapillary ependymoma from spinal ependymoma

Suvendu Purkait[1,2], Sophia Praeger[3,4], Jörg Felsberg[2], David Pauck[2], Kerstin Kaulich[2,5], Marietta Wolter[2], David Koppstein[3,4], Guido Reifenberger[2,5]

[1] Department of Pathology and Laboratory Medicine, All India Institute of Medical Sciences, Bhubaneswar, Odisha, India
[2] Institute of Neuropathology, Heinrich Heine University Medical Faculty and University Hospital Düsseldorf, Düsseldorf, Germany
[3] Cancer Bioinformatics and Multiomics (ED08), German Cancer Research Center Heidelberg and German Cancer Consortium (DKTK), partner site Essen/Düsseldorf, Düsseldorf, Germany
[4] Department of Pediatric Oncology, Hematology and Clinical Immunology, Heinrich Heine University Medical Faculty and University Hospital Düsseldorf, Düsseldorf, Germany
[5] German Cancer Consortium (DKTK), partner site Essen/Düsseldorf, Düsseldorf, Germany

SUPPLEMENTAL MATERIAL

SUPPLEMENTAL FIGURES

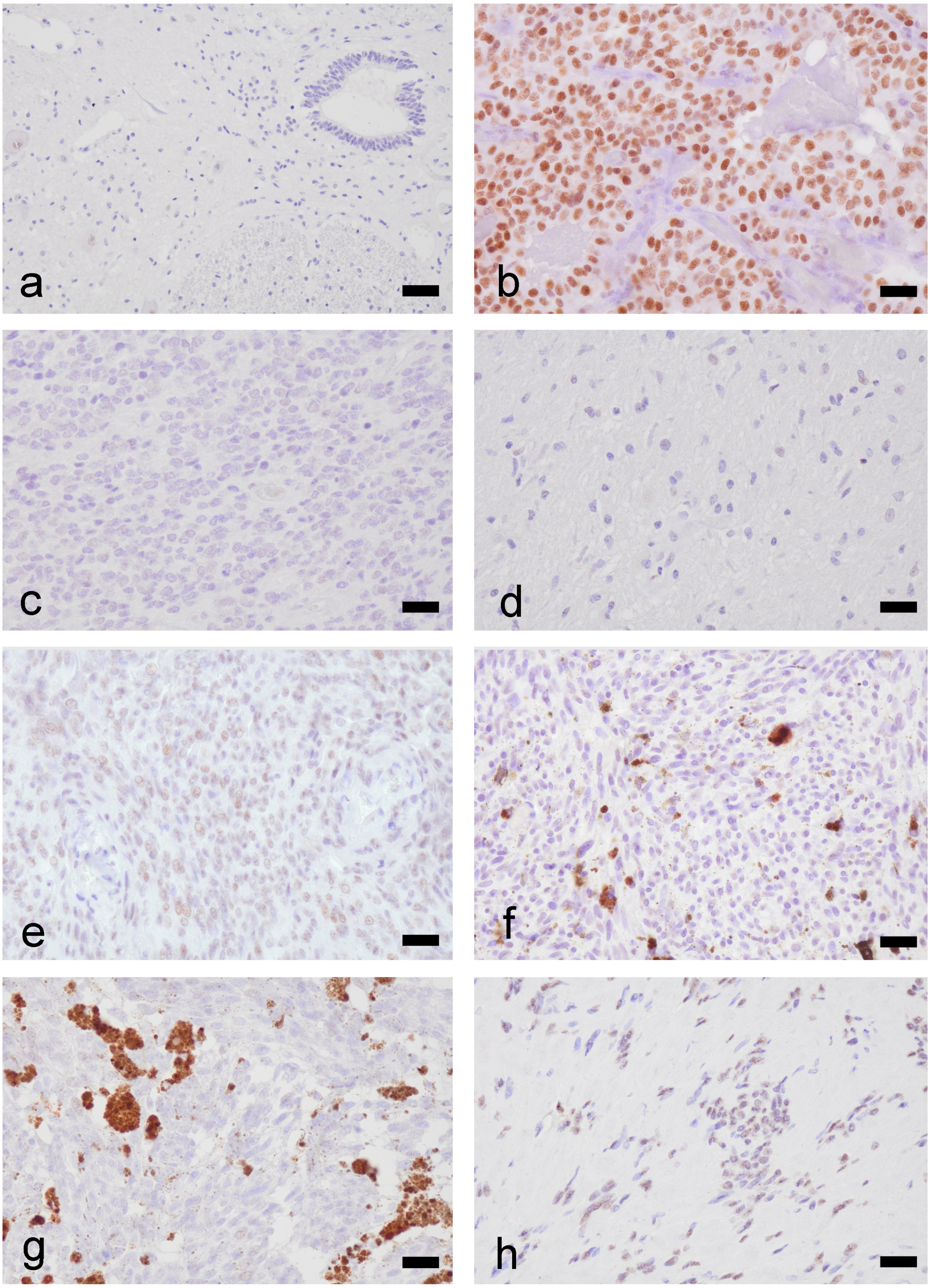

Supplemental Figure 1. Exemplary immunohistochemical stainings for HOXB13 in normal spinal cord (**a**) and various types of non-ependymal tumors of the spinal cord **(b-h**). Immunostaining for HOXB13 in adult spinal cord tissue remained negative (**a**). In contrast, immunostaining of a paraganglioma of the cauda equina region revealed

strong nuclear expression of HOXB13 (**b**). Other spinal tumor types including diffuse midline glioma, H3 K27-altered (**c**), pilocytic astrocytoma (**d**), meningioma (**e**), melanocytic tumor of intermediate differentiation (**f**), melanotic peripheral nerve sheath tumor (**g**) and schwannoma (**h**) remained negative for HOXB13. Sections are immunohistochemically stained with the anti-HOXB13 antibody (brown) and counterstained with hemalum (blue). The brown structures in (f) and (g) correspond to melanin pigment. Scale bars correspond to 50 μm.

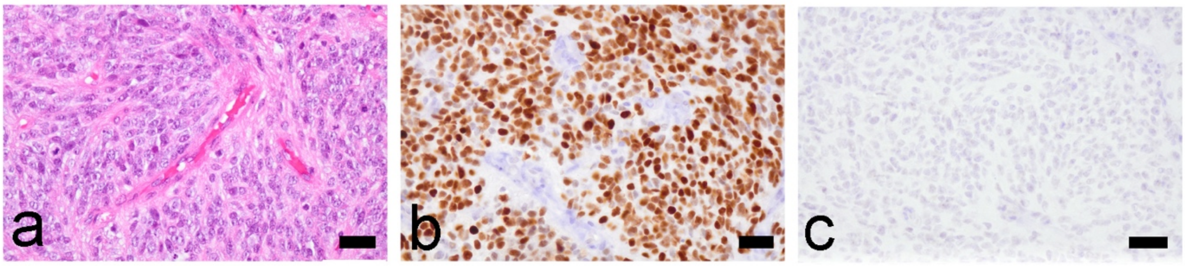

Supplemental Figure 2. Exemplary histological and immunohistochemical findings in a case of spinal ependymoma, *MYCN*-amplified (case AE81). (a) Hematoxylin-eosin staining shows a cellular ependymal tumors with formation of perivascular pseudorosettes. (b) Immunohistochemical staining for MYCN demonstrates strong nuclear expression corresponding to *MYCN* gene amplification in the tumor cells as detected by ddPCR analysis (not shown). (c) Immunohistochemical staining for HOXB13 remained negative. Scale bars correspond to 50µm.

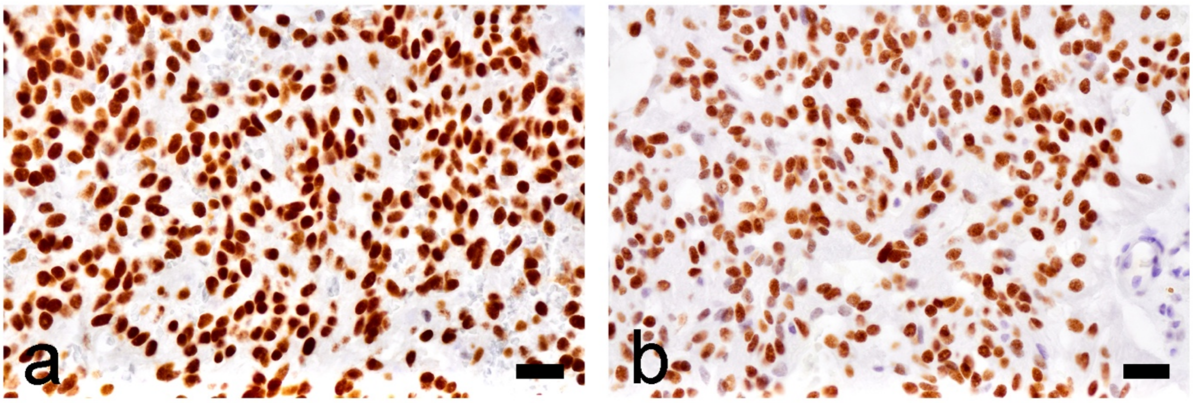

Supplemental Figure 3. Strong nuclear staining for HOXB13 in a myxopapillary epepndymoma (case MPE90) after (a) 1 day or (b) 6 weeks of fixation in 4% buffered formalin. Note that prolonged fixation did not change strong nuclear HOXB13 positivity. Scale bars correspond to 50µm.

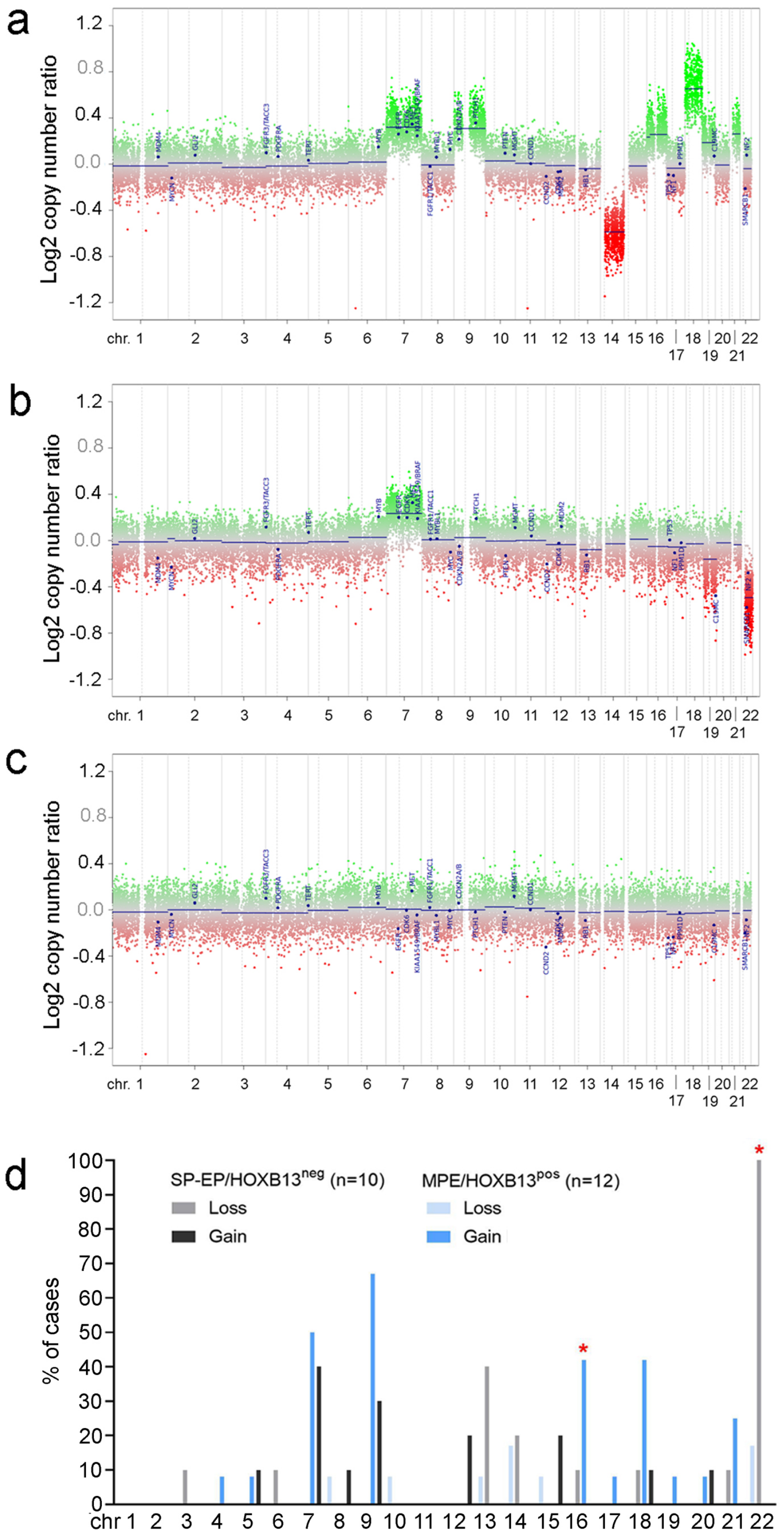

Supplemental Figure 4. DNA copy number profiles of individual cases of MPE (case MPE79) (**a**), SP-EP (case E175) (**b**) and SP-SE (case SE78) (**c**). Note multiple copy number gains and losses in the MPE (a), chromosome arm 22q loss in the SP-EP (b), and no copy number alterations in the SP-SE (c). (**d**) Comparison of the frequency of copy number alterations on each chromosome in MPE versus SP-EP. L, copy number

loss; G, copy number gain. *Note more frequent loss of 22q in SP-SE ($p=0.0001$, Fisher's exact test) and more frequent gains on 16 in MPE ($p<0.05$, Fisher's exact test). Only a single MPE showed a low-level copy number gain of chromosome 17 encompassing the *HOXB13* gene locus.

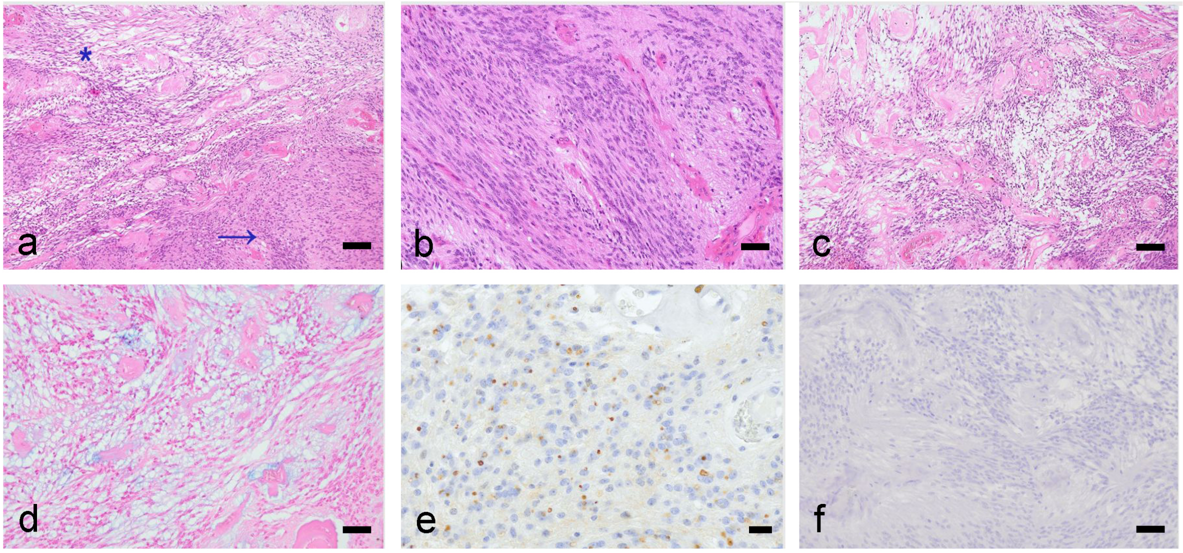

Supplemental Figure 5. A representative case of spinal ependymal tumor (case E169) with myxoid area (star) and solid areas (arrow) (**a**). The solid area showed perivascular pseudorosettes (**b**), while the myxoid area showed papillary arrangement with vascular hyalinization (**c**), and significant myxoid change upon alcian blue staining (**d**). Immunostaining for EMA was positive in a dot-like manner (**e**), while immunostaining for HOXB13 remained negative (**f**). Scale bars correspond to 50 μm (b, d, e. f) or 100 μm (a, c).

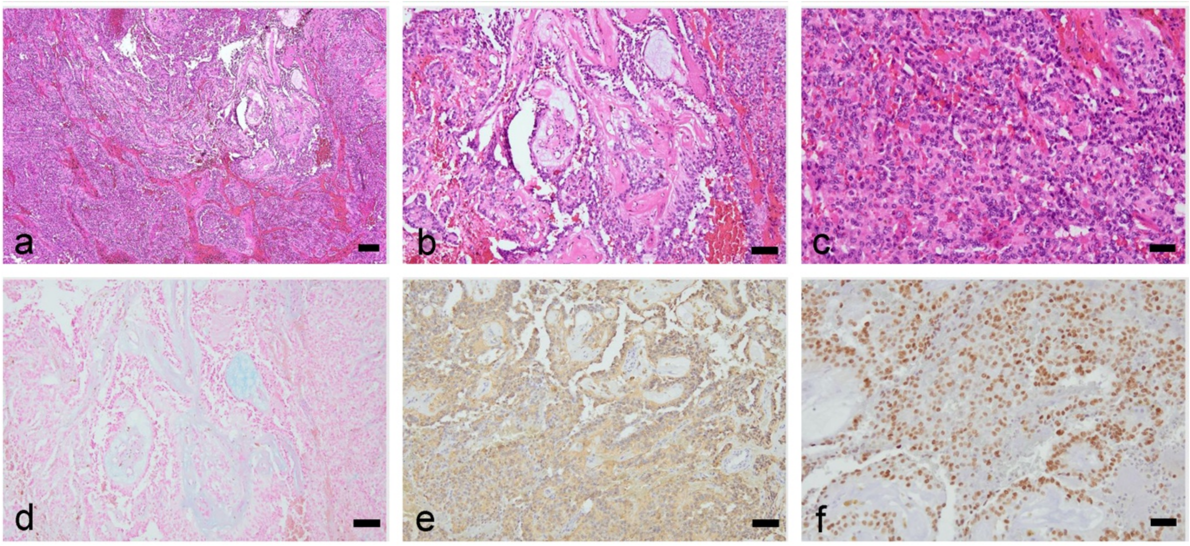

Supplemental Figure 6. A representative case of myxopapillary ependymoma (MPE62) with only occasional foci of papillary and myxoid areas but predominant solid areas (**a-c**). The myxoid areas are highlighted with alcian blue (**d**). Immunostaining for EMA was weakly and diffusely positive (**e**), while the tumor cell nuclei were strongly positive for HOXB13 (**f**). Scale bars correspond to 200 μm (a,d), 100 μm (b, e) and 50 μm (c, f).

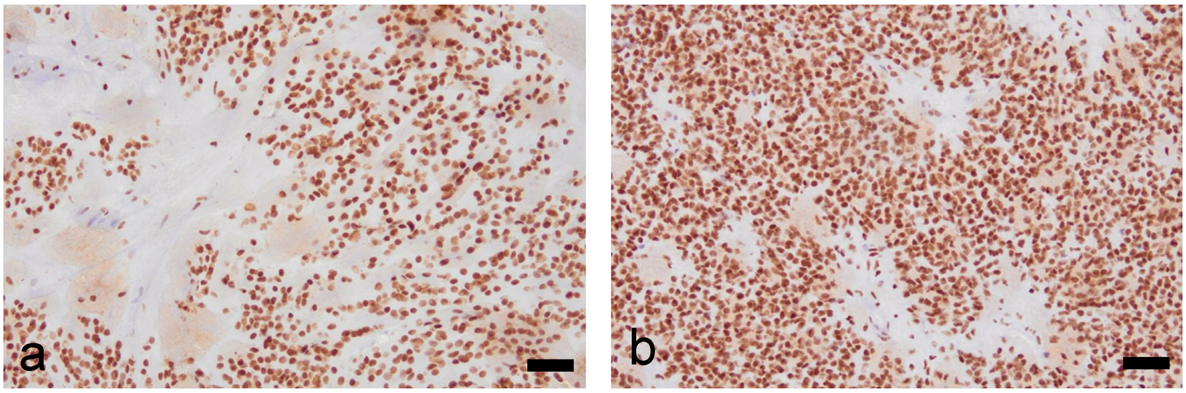

Supplemental Figure 7. Immunohistochemistry for BRD4 in a selected case of MPE (case MPE65) (**a**) and SP-EP (case E143) (**b**). Note that neoplastic cells in both tumors showed strong and uniform nuclear immunoreactivity for BRD4. Sections are immunohistochemically stained with the anti-BRD4 antibody (brown) and slightly counterstained with hemalum (blue). Scale bars correspond to 50 μm.

SUPPLEMENTAL TABLES

Supplemental Table 1. HOXB13 immunohistochemical expression in spinal ependymal and non-ependymal tumors, and adult spinal cord tissue.

Histological diagnosis	HOXB13 nuclear expression		
	Strong	Weak	Negative
Myxopapillary ependymoma (CNS WHO grade 2)*	54	0	0
Spinal ependymoma (CNS WHO grade 2)*	11	10	25
Spinal subependymoma (CNS WHO grade 1)	0	3	2
Spinal ependymoma, *MYCN*-amplified	0	0	2
Spinal metastases of PFA/B ependymomas	0	0	2
Spinal diffuse midline glioma, H3K27-altered (CNS WHO grade 4)	0	0	3
Spinal glioblastoma, IDH-wildtype (CNS WHO grade 4)	0	0	1
Spinal pediatric-type diffuse high-grade glioma, H3-wildtype and IDH-wildtype (CNS WHO grade 4)	0	0	1
Spinal pilocytic astrocytoma (CNS WHO grade 1)	0	1	4
Spinal meningiomas (CNS WHO grade 1 or 2)	0	4	2
Spinal meningeal melanocytoma / melanocytic tumor of intermediate differentiation	0	0	4
Spinal melanotic peripheral nerve sheath tumor	0	0	1
Spinal schwannoma or neurofibroma, (CNS WHO grade 1)	0	3	3
Cauda equina neuroendocrine tumor (previouslsly paraganglioma (CNS WHO grade 1)	5	0	0
Adult spinal cord	0	0	3

*As defined by histology according to WHO 2021 criteria.

Supplemental Table 2. Details of the cases subjected to DNA methylation profiling and next generation gene panel sequencing.

Case-ID	Initial diagnosis	CNS WHO grade	Special histologic features	HOXB13 IHC	Methylation class	Calibrated classifier score	DNA copy number losses	DNA copy number gains	DNA sequence variants
E162	SP-EP	2	Focal myxoid areas	Strong	MPE	0.99	22q	-	-
E52	SP-EP	2	Focal myxoid areas	Strong	MPE	0.99	-	7, 9, 16	-
E134	SP-EP	2	Focal myxoid areas	Strong	MPE	0.99	-	9	-
E102	SP-EP	2	Focal myxoid areas	Strong	MPE	0.99	8, 10, 13q, 14q, 15q	7, 9, 16, 18, 21q	-
E176	SP-EP	2	-	Strong	MPE	0.99	-	-	-
E149	SP-EP	2	-	Strong	MPE	0.99	-	-	-
MPE41	MPE	2	Solid areas	Strong	MPE	0.99	-	7, 9, 16, 18, 20, 21	*EGFR*: NM_005228.5: exon3:c.379G>A: p.Ala127Thr
MPE65	MPE	2	Solid areas	Strong	MPE	0.99	-	-	-
MPE86	MPE	2	Solid areas	Strong	MPE	0.99	22q	7, 9	-
MPE79	MPE	2	Solid areas	Strong	MPE	0.99	14	7, 9, 16, 18, 19, 21	-
MPE62	MPE	2	Solid areas	Strong	Ependymal tumors/MPE	0.52/ 0.35	-	4, 9, 18	-
MPE82	MPE	2	-	Strong	MPE	0.99	-	5, 7, 9, 16, 17, 18	-
E177	SP-EP	2	Focal myxoid areas	Weak	SP-EP	0.99	3, 14q, 16, 18 22q	-	-
E175	SP-EP	2	-	Weak	SP-EP	0.99	22q	7	*NF2:* NM_000268.4: exon7: c.675+1G>C:p.?
E170	SP-EP	2	-	Weak	SP-EP	0.99	22q	7, 9, 12	-
E158	SP-EP	2	-	Weak	SP-EP	0.99	13q, 14q, 21q, 22q	-	-
E173	SP-EP	2	-	Weak	SP-EP	0.99	22q	5, 7, 8, 9, 12, 15q, 20	-
E150	SP-EP	2	-	Weak	Ependymal tumors/ SP-EP	0.37/0.17	13q, 22q	-	*NF2*: NM_000268.4: exon6: c. 592C>T: p.Arg198Ter
E51	SP-EP	2	Focal myxoid areas	Negative	SP-EP	0.99	13q, 22q	-	-

E145	SP-EP	2	Focal myxoid areas	Negative	SP-EP	0.99	6q, 13q, 22q	-	-
E178	SP-EP	2	-	Negative	SP-EP	0.99	22q	7, 9, 15q,18	-
E174	SP-EP	2	-	Negative	SP-EP	0.99	22q	-	*RB1*: NM_000321.2: exon17: c.1573G>A: p.Ala525Thr
AE80	PFB-EP*	3	-	Negative	PFB-EP	0.99	6	15q, 18q	-
AE54	PFB-EP*	3	Focal myxoid areas	Negative	PFB-EP	0.87	22q	1q, 4, 5, 7, 8, 9, 12, 18, 19, 20	-
AE21	PFB-EP*	3	-	Negative	Ependymal tumors/ PFB-EP	0.49/0.15	2p, 6q, 17p, 22q	7, 8, 17q	-
AE22	PFA-EP*	3	-	Negative	Ependymal tumors/ PFA-EP	0.56/0.35	6q	1q, 7, 8, 9, 14q, 17, 19, 20, 21q	-
SE111	SP-SE	1	-	Weak	SP-SE	0.99	-	-	-
SE110	SP-SE	1	-	Weak	Ependymal tumors/ SP-SE	0.38/0.11	3, 9	-	*TERT:* NC_000005.9: g.1295228G>A
SE78	SP-SE	1	-	Negative	SP-SE	0.99	-	-	-

*Spinal metastases of posterior fossa ependymomas: n.d., not determined due to poor quality of the DNA copy number profile
